# The eGenVar data management system—cataloguing and sharing sensitive data and metadata for the life sciences

**DOI:** 10.1093/database/bau027

**Published:** 2014-03-28

**Authors:** Sabry Razick, Rok Močnik, Laurent F. Thomas, Einar Ryeng, Finn Drabløs, Pål Sætrom

**Affiliations:** ^1^Department of Cancer Research and Molecular Medicine, Norwegian University of Science and Technology, Prinsesse Kristinasgt. 1, NO-7491 Trondheim, Norway and ^2^Department of Computer and Information Science, Norwegian University of Science and Technology, Sem Sælands vei 9, NO-7491 Trondheim, Norway

## Abstract

Systematic data management and controlled data sharing aim at increasing reproducibility, reducing redundancy in work, and providing a way to efficiently locate complementing or contradicting information. One method of achieving this is collecting data in a central repository or in a location that is part of a federated system and providing interfaces to the data. However, certain data, such as data from biobanks or clinical studies, may, for legal and privacy reasons, often not be stored in public repositories. Instead, we describe a metadata cataloguing system and a software suite for reporting the presence of data from the life sciences domain. The system stores three types of metadata: file information, file provenance and data lineage, and content descriptions. Our software suite includes both graphical and command line interfaces that allow users to report and tag files with these different metadata types. Importantly, the files remain in their original locations with their existing access-control mechanisms in place, while our system provides descriptions of their contents and relationships. Our system and software suite thereby provide a common framework for cataloguing and sharing both public and private data.

**Database URL:** http://bigr.medisin.ntnu.no/data/eGenVar/

## Introduction

Storing data for future reuse and reference has been a critical factor in the success of modern biomedical sciences and has resulted in several landmarks, such as the UniProt (1) and GenBank (2) collections of protein and nucleotide sequences. Motivations for these efforts include providing proper references to data discussed in publications and allowing published data to be easily reused for new discoveries. This way, proteins with known functions have, for example, been used to infer properties of newly discovered genes. Similarly, more recent efforts, such as the encyclopedia of DNA elements (ENCODE) (3), 1000 genomes project (4) and cancer genome projects (5), are reused and combined to uncover links between, for example, chromatin structure and mutation rates (6) or common disease-associated DNA variants and regulatory regions (7).

Another motivation for storing data is reproducibility: having data and information on how the data were produced and analysed—so-called metadata—enables replication studies. Importantly, the additional metadata is critical for reproducibility (8). Although open archives such as GenBank (2) and ArrayExpress (9) are invaluable as tools to promote free reuse of published data, open archives bring risks of exposing private information about study subjects. By using data from freely available genealogy databases, Gymrek and colleagues could, for example, identify the surnames of several participants in the 1000 genomes project (10). Current archives for individual genetic data, such as microarrays used in genome-wide association studies (GWAS), therefore have access-control mechanisms that typically only allow access to pre-approved studies (11). Nevertheless, whereas access to genetic data should be controlled because of privacy concerns, the metadata that describe the technical aspects of experiments and analyses can be freely shared. Indeed, sharing technical data such as the microarray platform used, the phenotype studied and the number of subjects that participated in a GWAS study would not breach privacy but could facilitate reuse of the data in future studies.

The NCBI resource dbGaP (http://www.ncbi.nlm.nih.gov/gap), which is a central repository for genotype and phenotype data, extracts such metadata from submitted datasets and provides an interface to search through them. Data need to be submitted to dbGaP before the metadata are extracted and access restrictions can be imposed on uploaded files. Similarly, a locally installable web-based resource that uses metadata to find and share data is the SEEK software (12). In the SEEK system the metadata are extracted from the data included in the system and organized using an approach based on the ISA tools (13). One common feature of these systems is that the files need to be added to the system and then some form of data extraction takes place. This may lead to requiring additional authorization and security precautions when working with sensitive data. Therefore, a system that does not access the file content directly and collects only the metadata required to advertise their presence would be a valuable resource.

Metadata relevant to files generated in the life science domain can broadly be grouped into three classes: information about files, file contents or relationships between files. File information includes details such as file name, owner, date created and location path. These context-independent metadata can be obtained directly from the operating system or associated tools (such as the Unix command **stat**), are valid across different computational platforms and file types, and are generally useful across scientific domains. However, to interpret data, users require domain-specific details about file content (second class of metadata). For biomedical data, for example, attributes like experimental protocols followed, technologies used, disease conditions investigated and genes involved could all be necessary to make the data meaningful. Ontologies and controlled vocabularies are approaches to make this process of storing domain-specific file content metadata systematic and standardized. Moreover, the biomedical research community has developed standards that describe the minimum information that should be present for data generated by different technologies to be considered reusable and reproducible (14). Although some of these standards are collections of general titles rather than descriptive checklists, others, such as the Minimum information about a microarray experiment (MIAME) (15) are actively used by data repositories, such as ArrayExpress (16), to guide the data submission process.

The third important aspect of data management is the relationships between different data files or entities (third class of metadata). Such relationships include both sample information—for example, that two files are generated from the same or related biological samples—and process information or provenance—for example, that a result file was generated by using a specific program and auxiliary data to analyse a set of data files. In general, maintaining such hierarchical relationships is more complex than keeping related files in project-specific folders and sub-folders. To illustrate, some relationships, including inbreeding, can only be described by a directed acyclic graph (DAG), which excludes tree-based file systems from being used (Supplementary Figure S1). Moreover, strictly using file system placement to represent relationships would often require altering files; for example, a file that originally contained data on 1000 individuals would have to be split into 1000 separate files and placed in a structure that reflects file and sample relationships.

Scientific workflow management systems, such as Galaxy (17) or Taverna (18), use DAGs to model and record tasks and dependencies. Although these workflows are primarily used to achieve reproducibility, they could also be used to determine data required, processing steps, order of processing and software used at each step. Workflow systems also provide a way to share data, as others with access rights can use the data included in the system. Whereas these systems excel in what they are designed to do, workflow systems have several limitations if used as a general data management system.

- Data provenance is only available from the point where the data were introduced to the system and only for the processing recorded in the workflow structure. For example, raw data that were modified before being introduced to the system, processing steps performed by proprietary software such as GenomeStudio (Illumina Inc.), or original versions of anonymized data from a clinical study, would not be available through such a system.
- Data exported from the workflow system loses its provenance. Such exports could, for example, be necessary when using novel methods to process data.
- Relationships between data introduced or present in the system, such as data from similar or related samples, are not readily part of data analysis workflows, but have to be recorded in separate systems such as Laboratory Information Management Systems (LIMS).
- Data must be moved from their original locations to be advertised and shared through workflow systems. But data containing sensitive information may have legal restrictions or requirements for added security that cannot be met by standard workflow systems.
- File handling is opaque such that file names and locations are only meaningful within the system. Uploaded files may, for example, be internally renamed and processing results may be stored in a system-defined structure, so that data cannot be located without using the workflow system.

The Synapse project management system (https://www.synapse.org/) by Sage software is a commercial workflow system that has addressed some of these limitations by providing data sharing options for local and distributed data. Data owners can share data through Synapse by making the data public or shared with a team of specific users, put private data remain hidden so that the system cannot be used to advertise such data.

Although having detailed metadata on content, provenance and sample relations enables both replication and reuse, thereby increasing the data’s scientific impact, scientists will in reality weigh these benefits against the labour required to report and maintain the metadata. Clearly, existing information should be easily reusable without having to provide them again. Moreover, inherent relationships should be captured and the origin and progression through time should be recorded as it happens. Further, metadata reporting should be possible in stages with the involvement of different people and without requesting all details upfront. Free text descriptions of content should be possible, but relevant ontologies and controlled vocabularies should be available and used for structuring reporting and maintaining uniformity. Additionally, data providers having sensitive and private data should be able to report the presence of the data without violating ethical or legal requirements by exposing the data itself. Finally, the reporting process should be possible as part of routine work and not something left until there is an obligation to do so.

At the same time, the technologies and methods used in research are becoming more advanced and increasing in number. This increases the complexity and quantity of the data produced, which tend to take a variety of formats. In addition, lack of a central catalogue for research data, inability to host sensitive data on public servers, and privacy and security concerns make it hard to locate existing data leading to redundancy in research and ambiguity in resources used. These issues have been discussed in initiatives like the Big Data to Knowledge program (BD2K) (http://commonfund.nih.gov/bd2k/). Specifically, BD2K suggests that a resource that stores research data and metadata will be as valuable as PubMed is for publications. Currently, the European life sciences Infrastructure for biological Information (ELIXIR) (http://www.elixir-europe.org/) project is working on a coordinated infrastructure for sharing and storing research data from the life sciences. This effort brings an opportunity to catalogue the vast amount of data collected from many sources; the challenge is to create systematic methods and standard tools to achieve this task effectively and efficiently.

A data catalogue with content summary information would be a very useful for reporting the presence of sensitive data as well. As a specific example, for biobanks, data reproducibility and reuse are important, as the banks typically have limited quantities of biological samples such as blood, saliva or urine, and acquiring new samples is costly or infeasible. At the same time, data produced from such samples such as gene expression or genetic variation measurements, can potentially be reused to investigate other phenotypes than the study that produced the original data investigated. Consequently, both the biobank’s phenotypic diversity and its ability to track data content, provenance and sample metadata are critical factors for the biobank’s scientific impact.

**Figure 1.**
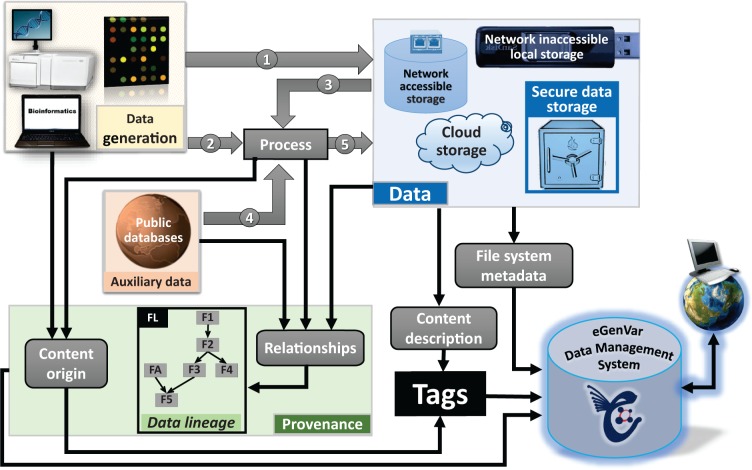
Types of metadata handled by EGDMS. Data originated from wet-lab experiments or computational analysis can both be raw data (1). Raw data subjected to processing become processed data (5). Processed data can then be raw data to subsequent processing steps (3). Provenance captures how data originated and the processing steps required in reaching their current state. Data lineage records the hierarchical arrangement of the entities by considering the entities required to generate them (parents) and their involvement in producing other entities (children). The box FL shows how this information can be represented as a graph (see Figure 3 for more on details on how the box FL is derived using relationships). Processing may require auxiliary data from public databases (4). Such auxiliary data include resources like a reference genome, gene annotation, or data from instrument and reagent manufactures. This auxiliary data are a part of the data lineage information, although it may not be stored with the other data. Data can reside in common secure storage, storage accessible via network including cloud storage solutions, local storage not connected to the network, or in external storage, devices (e.g. tape archives, removable disks). File system metadata such as location and owner are directly acquired by the EGDMS system. Provenance and content description are included as desensitized tags.

This paper describes a method and a software suite for sharing information without compromising privacy or security. This system constructs a metadata catalogue, which could be used to locate data while the original files remain in a secure location. Specifically, our system, called the eGenVar data-management system (EGDMS), allows users to report, track, and share information on data content, provenance and lineage of files (Figure 1). The system is designed to bridge the gap between current LIMS and workflow systems and to keep provenance for data being processed through disparate systems at different locations. Central to our system is a tagging process that allows users to tag data with new or pre-existing information, such as ontology terms or controlled vocabularies, at their convenience. The system is available as a self-contained installable software package that includes a server, command line client and a web portal interface. The system is lightweight and can be easily installed as a utility on top of existing file management systems. The main use-case for the EGDMS is handling and integrating anonymized biobank data, but the system has a flexible and extensible design to accommodate many other types of data used or generated in biological and medical research environments. In total, the EGDMS addresses one of the key challenges in current scientific resource and data management: allowing flexible annotation, sharing and integration of public and private biomedical research data.

## Results

The following sections describe our solution for managing metadata: the EGDMS. We first define the terminology we use to describe different aspects of our system and the specific metadata our system handles. We then describe our implementation, how EGDMS manages users and access control, and the processes of depositing data, handling errors, updating and synchronizing data, and retrieving data. Finally, we provide a case study on how EGDMS can manage metadata on diverse GWAS studies in a biobank setting.

### Terminology

The ISO 15489-1 documentation (19) defines metadata as data describing record context, content, structure and management through time. For example, data files have name, creation date and ownership information as metadata. In addition to such file system metadata, which can be obtained directly from the operating system, this article considers *provenance*, *data lineage* and *content description *metadata, which are described in detail under metadata extensions.

**Figure 2.**
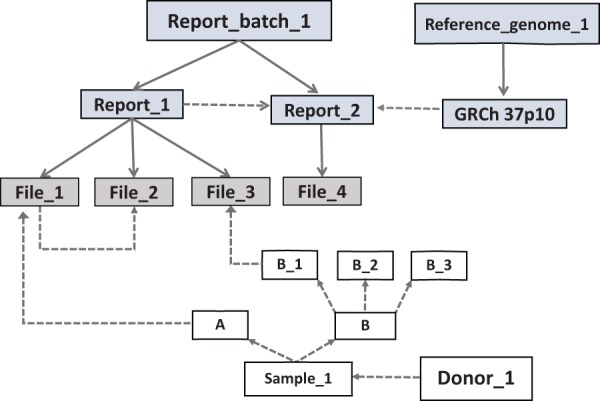
The basic arrangement of an EGDMS record. Files are grouped under reports and related reports are grouped under report batches. The source of the samples is the donor. Solid lines show group membership information with arrowheads pointing from the group title to the members. Dashed lines show data lineage relationships with arrowheads pointing from parents to children. The files File_1 and File_3 contain results from the same experiment; therefore, they are grouped under the same report (Report_1). The experiment was conducted using samples A and B_1, which originated from the donor Donor_1. File_2 is a normalized version of File_1 and does not have a direct link to a sample, but it is linked as a child to File_2 and grouped under the same report. This arrangement extends the relationships of File_1 to File_2. Report_2 contains the results (File_4) of a bioinformatics analysis conducted using the files in the Report_1. Therefore, Report_2 is a child of Report_1. Report_2 uses a reference genome connected to a report (GRCh 37p10) in the report batch called Reference_genome_1 (file level details not shown). *Note*: grey boxes represent data and white boxes represent samples.

File system metadata can be obtained from basic system services; for example, through the UNIX systems call **stat** or the Apache Commons Net library™ (http://commons.apache.org/proper/commons-net/). In our system, the captured metadata translate into *entities *and relationships between them. An *entity* is the database representation of a real-world object or a concept with a unique set of attributes (20) . For example, details about files are captured by the database entity *files*. The set of metadata about a file is a *record*. The person creating such a *record* is a *reporter.* The *files* are organized in a research project, publication or an experiment-based criteria known as a *report*. Related *report*s are grouped under a *report_batch *(Figure 2). The *files* can be *raw data, processed data* or *auxiliary data*. *Raw data* are generated from experiments to start with and operations on it produce *processed data*. The *processed data* when used as input in subsequent processing become *raw data* to that step. Data from public databases, reference genomes, data sheets and product identification sheets from instrument or reagent manufactures are some examples of *auxiliary data *(Figure 1).

### Metadata extensions

**Figure 3.**
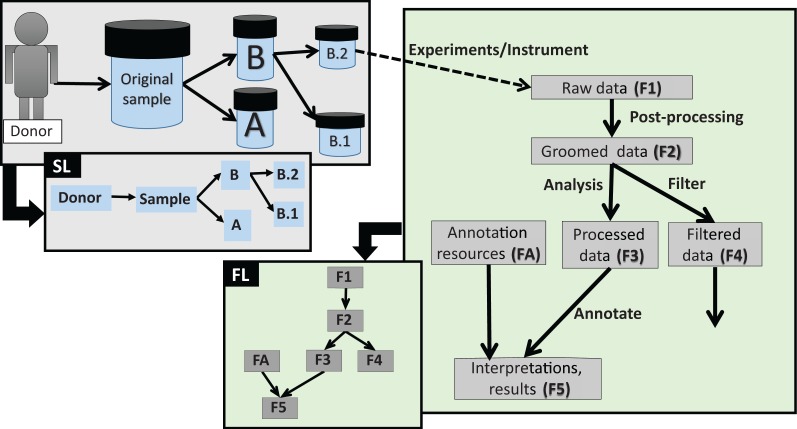
Different types of data lineage information captured by EGDMS. Box SL shows relationships between a donor, the original sample and sub-samples. As the sample was obtained from the donor and samples B.1 and B.2 are aliquots of the original sample, this is a natural existing relationship. Box FL shows relationships between files processed through a series of analyses. The data in the groomed data file (F2) is derived from the raw data files (F1). The annotation resources (FA) are used when generating file F5 from file F3. When this information is recorded as provenance, data lineage can be constructed as shown in the figure. Solid lines indicate the direction of the flow, where the arrow points from parent to child. Dashed lines indicate the link between the samples and the data generated using them.

We use three extensions to the basic file system metadata for effectively describing data from the life sciences domain (Figure 1). *Provenance* describes how the data originated and the processing steps required in reaching the current state. For example, the details about the instruments used and protocols followed during an experiment becomes *provenance* information for the data produced from that experiment. The relationship information manifested between data, when the *provenance* is fully recorded is the *data lineage* (Figure 3). The *data lineage* is a hierarchical arrangement of the entities considering the parent entities required to generate them and their involvement in producing other entities (children). For example, the donor is the parent of the samples and a normalized version of a file is a child of that file. The *content description *describes the content of a file by using tags, constructed using controlled vocabularies and other terms relevant to describing the content. Controlled vocabulary terms are acquired from public resources, which standardize the terms used in describing experiments and results. The Open Biological and Biomedical Ontologies (OBO) (http://obofoundry.org/) and the phenotypic details of the content such as disease conditions investigated are an example of *content descriptions*.

### Implementation

**Figure 4.**
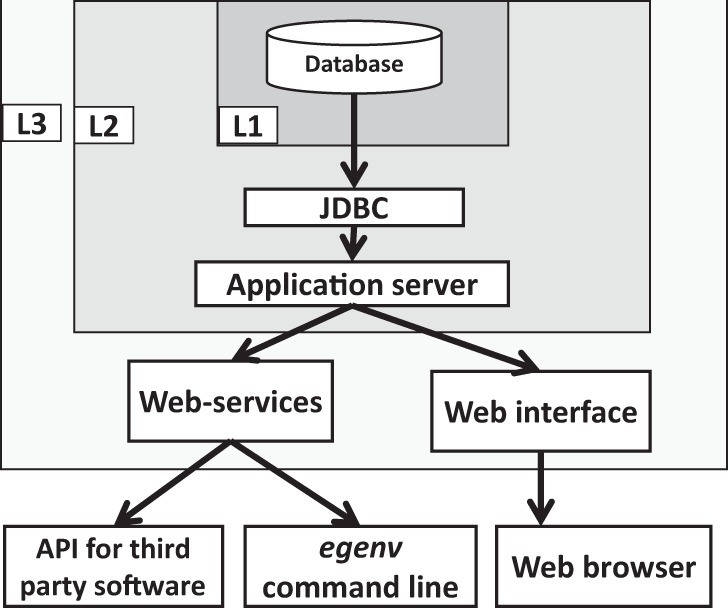
The basic components, their connectivity and the security levels of the EGDMS. Embedded JavaDB is the default DBMS and resides in the level one (L1) security container. The database is configured in non-network accessible mode and the users cannot directly interact with the database. The application server is in level two (L2) security and communicates with L1 using the JDBC API and maintains a connection pool for the applications to access. The application server should be started by the same process as the database server and requires a password to connect to the database server. The application server limits access by requesting a user name and a password. It also manages user roles (edit, search only etc.). The level three (L3) security is currently not implemented by the EGDMS, and is part of the server hosting it and represents a system firewall. The web-services and the web interface are accessible from the open ports of the host server. The egenv tool with a web-service client resides outside the security barriers and requires authentication to access the resources.

The EGDMS is developed using Oracle® Java enterprise software (Java EE) (http://www.oracle.com/technetwork/java/javaee/) and consists of a server and a command line and a web interface clients. The server uses a relational database for user account management and data storage. The communication between server and the database is achieved using Java Database Connectivity API (JDBC) (Figure 4). The command line tool uses web-services to connect to the server. The default database management system (DBMS) embedded in the system is Java DB (http://www.oracle.com/technetwork/java/javadb/) which is Oracle's supported distribution of the Apache Derby (http://db.apache.org/derby/) open source database. Alternatively, dedicated database servers such as MySQL (http://www.mysql.com/) could be used with the system for faster, high volume operations using the connectors provided. The default application server is the embedded version of Sun Java Application server code named Glassfish.

The command line tool and web interface cover two different ranges of users. The UNIX command line tool *egenv* covers one extreme where the user invokes commands from the UNIX terminal. Using this tool requires basic competence with the UNIX terminal operations, but allows experienced users script-based access to EGDMS. The other extreme offers a user-friendly graphical user interface, invoked from a web browser. The web-services expose an application-programming interface (API), which third party software can use to communicate with the system. The users are free to use one interface or go between different ones to complete different tasks. This approach allows users with different levels of competence to achieve the same set of tasks.

The EGDMS software suite and the programming code described here are available for download from http://bigr.medisin.ntnu.no/data/eGenVar/. All products developed by the authors are free of charge and distributed under the Creative Commons Attribution 2.5 Generic license (http://creativecommons.org/licenses/by/2.5/). The third party components that the system depends on carry their own licence agreements, but these generally follow free and open source requirements and are described in detail in the documentation. Detailed system requirements, installation instructions and the client usage tutorials also available from the above link.

### Managing users and access control

Users need to have an account to access the system. Users can associate search, data entry, edit and update privileges to their accounts during registration. By default, user verification uses the email address provided during registration. Optionally, two-factor authentication can be used during registration if the system is configured to use a short message service (SMS).

Users can login and use the web interface once their user accounts are activated. For the command line tool, users need to register the tool’s access location with the web-service by obtaining a non-transferable certificate. The term non-transferable means that each user needs to obtain an individual certificate for each installation of the tool. Once authenticated, the tool can be used from the same system user account without any other login requirements as long as the certificate is valid. Authenticated users can immediately start using the system for search operations. However, a personal profile needs to be created before adding, deleting or updating content. The system is compatible with additional security layers, such as firewalls based on iptables (http://www.netfilter.org/) rules, but the EGDMS does not provide its own firewall configurations, as all the installation tasks are designed for users without administrator privileges and to maintain cross platform compatibility.

### Depositing data

Data depositing is the process of reporting the presence of a file and attaching all relevant meta-information with it. This process has three steps.

1. Create records.
2. Attach *data lineage*.
3. Tag records.

The three steps can be executed simultaneously or at different stages, involving one or more depositors. The reported files are organized in *reports* and *report batches *(Figure 2). By default, the system uses folder names to infer this arrangement. Specifically, when invoked on a file inside a folder, the system considers all files inside the folder to be in the same *report* and the parent folder will designate the *report_batch*. Note that relative paths are used when deciding *report *and *report_batch *names while the absolute path of the files are recorded. Consequently, files that logically belong to the same report but are distributed in different sub-folders or on different servers can automatically be added to the same report as long as the names of the folders and parent folders are identical at the different locations or such arrangement is simulated using virtual links. Alternatively, the depositor can construct this arrangement by manually specifying the names for *reports* and *report_batches *during the depositing process. In addition to this grouping, the file system metadata of the files, the checksum values, last modified date and ownership information are recorded during the depositing process. For the command line tool, issuing the command **egenv –prepare** creates a set of configuration files with the above details for the current directory and **egenv –add** adds the details to the database. These configuration files can be edited or alternatively generated using third party programs before adding the details to the database. For the web interface, the depositor connects to the desired location through a secure connection, such as secure FTP, and selects the files to be added. EGDMS automatically creates a record with the structure as explained above, and a set of default values, which the depositor or others can edit subsequently if needed.

After a record is created, *data lineage* information must be added. This information, which includes sample relationships and relationship between files, cannot be automatically extracted from file system metadata and must therefore be provided by the depositor. The *egenv* command line tool creates a template that can be filled in manually or automatically by, for example, using scripts to process sample sheets. In the web interface, *data lineage* can be added in batch mode or single record mode. The batch mode can add a child or a parent to multiple entities at a time, whereas the single mode adds one relationship at a time by selecting two entities and their relationship. Creating records and attaching *data lineage *information are the only compulsory steps of the depositing process. The two steps can be quickly accomplished by relying on the default values provided by the system; however, the system’s usefulness depends on the extent depositors provide additional information by customizing these default values and by tagging the submitted records.

**Figure 5.**
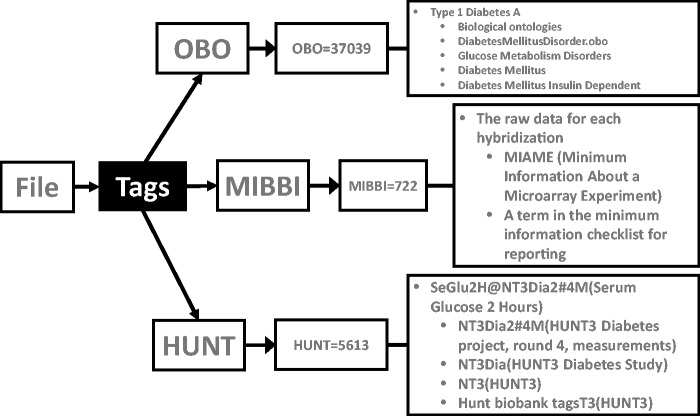
Tagging files. The file shown here has three tags: two from public databases (OBO = Open Biological and Biomedical Ontologies, MIBBI = Minimum Information about a Biomedical or Biological Investigation) and one from a private source (HUNT = The Nord-Trøndelag health study). The OBO tag OBO = 37 039 refers to the term ‘Type 1 Diabetes A’ and is an internal identifier used to ensure persistence between different OBO versions. As the OBO has a hierarchical arrangement, this file will be connected to other files describing ‘Type 1 Diabetes A’ and to, for example, files describing other types of glucose metabolism disorders. The MIBBI = 722 tag tells that the file contains raw data for each hybridization of a microarray experiment. The information about the microarray used, protocols followed etc., will also be attached to this file this way (not show for simplicity). The HUNT = 5613, tells that this file is related to the diabetes study of the phase three Hunt project, specifically ‘Serum Glucose 2 Hours’ values.

The EGDMS uses tags based on controlled vocabularies and standardized terms to attach provenance information and to describe file contents (Figure 5). The controlled vocabulary includes details obtained from OBO. The standardized terms come from a wide variety of sources including the minimum information checklists from the MIBBI (Minimum Information about a Biomedical or Biological Investigation), array identifiers and internal user-provided identifiers. All the terms used for tagging are arranged in a hierarchical manner. This arrangement has three main benefits. First, individual tags can be made less verbose as they inherit properties of their parents. Second, users can create new tags under the existing ones to achieve higher granularity, making the process extensible. Third, existing and created tags can be located and reused by traversing the hierarchy.

When tagging, the system guides the depositor through a step-by-step process to create a tagging template by selecting the tags and the *entities *to be tagged. During this process, the choices made at each step affect subsequent steps resulting in a uniquely tailored tagging template. The controlled vocabularies and standardized terms help to guide this process, but also link the tagged entities to established public knowledge bases as well as with each other, thereby increasing the power of the search and retrieval process.

### Submitting data and handling errors

Duplicated files waste resources and are sources of inconsistencies. When creating file records, the *prepare* step therefore uses checksums to check for duplicated files already contained in the system. The system outputs the results of this step in a log file along with information on processing errors and warnings. The depositor can then handle any duplicates by removing them from the generated template, as only the files listed in the template are considered for processing in subsequent steps.

It is also possible to control the files added by specifying them during the add step. This option is useful when the depositor wants to exclude some files in the directory processed or wants to create multiple *reports* from files in the same directory. When adding, entities already in the database are ignored. Therefore, if there is an error during an add step that involves multiple files, the depositor can correct the error and rerun the same command without worrying about creating duplicates. At the same time, if maintaining multiple copies of files is required, then one of those files can be recorded in the EGDMS and multiple paths can be attached to it. Moreover, when adding a large number of entries at once, the process is automatically split into stages and *undo* points are created at the end of each stage. The *undo* or *delete *options can then be used to safely remove any erroneously added entries. The EGDMS interfaces, including the API, automatically handle database level constraints and populate tables in the correct order. Consequently, API users do not need to know the EGDMS database schema. This also makes it possible for a single template to have data ending up in multiple tables in the database.

### Updating and synchronizing data

Occasionally, metadata needs to be updated, for example, because a file is changed or relocated to a different folder or server, the file’s report should be changed, or a newly introduced tag should be attached. Whereas users can update reports and tags by following similar steps as when depositing data, the *egenv* tool provides a specific option for identifying and handling multiple moved files. Specifically, the **egenv -diff **option compares the details of the files in the current location against the database and creates a report of the changes if there are any. Any changes to records can then be updated in the database by using a template-based approach similar to the process of adding data. When this operation is performed on a specific location, it will use checksum values to locate the files already deposited in the system and create a template of the changes required in the database. Second, after potentially modifying the template, an **egenv -update** operation with the template as argument commits the changes to the EGDMS. As this process can be performed recursively, users can update all entries on an entire server at once. Alternatively, the **egenv –update –current**, will automatically commit changes with default values without any user intervention.

### Retrieving data

The EGDMS can provide users with the locations of deposited data, but as accessing the data may require separate user privileges, the data must be retrieved by the users themselves. These data locations can be retrieved by using the get-all method, by browsing the web server, or by using search queries, filters or scannable codes. The get-all feature provided with the command line tool retrieves all file entries of a selected entity; users can then filter the content and use third party software such as **scp** to retrieve the data. The web interface provides a browse feature that uses filters to go through data displayed in a tabular format without specifying a search query. As for searches, these can either be free text searches on specific database fields, such as files.name=experiment_122, or structured searches.

**Figure 6.**
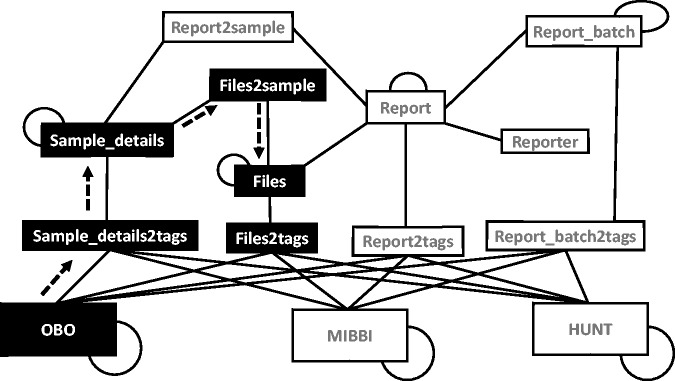
Graph arrangement of tables used in search operations. The graph is constructed using all the relevant tables (nodes) connected using connections (edges) constructed using foreign key constraints and polymorphic relationships. Self-loops indicate a hierarchical relationship. The black nodes and the dashed arrows show the path taken when answering the query ‘get all files connected to any of the samples tagged with the term Type 1 Diabetes A’. (OBO = The Open Biological and Biomedical Ontologies, MIBBI = Minimum Information about a Biomedical or Biological Investigation, HUNT = The Nord-Trødelag health study).

Structured search can involve many tables in the database at once (Figure 6). This is achieved by using a graph of all the relevant tables (nodes) connected through foreign key constraints and polymorphic association flags (edges). The polymorphic association flags are similar to foreign keys but could point to any one of the designated tables instead of just one. This technique is used in the tagging process, where a tag could be a link to any one of the controlled vocabulary, annotation or minimum information database tables. Still, users can construct queries just by knowing which group of properties (files, reports, samples and so on) they want to search. When a query is issued involving more than one table, the shortest path between them is used to retrieve the results. This graph-based navigation method makes it possible to construct dynamic queries, using fewer resources than when performing joins on tables.

Whereas searches can be done through both the command line and the web interface, the web interface provides an interactive method to refine search results. For example, once a result is obtained, the web interface uses the graph arrangement, grouping and the *data lineage* information to create links to additional details. The grouping of files in reports and reports in batches makes it possible to create queries like ‘get all members of the same group’. *Data lineage* is used to retrieve parents or children when that information is available; for example, the query ‘get all files used when generating the file file_0023’ relies on *data lineage *information.

Another search feature currently available with the web interface is to use scannable Quick Response (QR) codes to retrieve search results. Each search result generates a static URL and to repeat the search, this URL can be shared as a link or as a QR code. When shared as a QR code, any device with a QR decoding facility can reproduce the search results. In addition, all the sample and donor detail records have their own QR codes. This code can be used to label sample containers, for example, making it possible to use a code scanner such as a smart phone to retrieve sample details from the EGDMS.

### Case study

Although EGDMS is a general system for managing metadata, the system was specifically developed to handle the disparate data created from current biobank studies. Biobanks often provide samples, such as DNA, that collaborating research groups use to produce large-scale data on genetic variation. Although this genetic data may have initially been produced to study a specific phenotype, the data can potentially be reused in multiple future studies. As biobanks typically have limited quantities of biological samples, managing data produced by collaborating groups can become an important factor for a biobank’s future scientific impact.

One such example is the HUNT Study and Biobank, which has a rich set of phenotypes and stores human biological samples from ∼120 000 people in Nord-Trøndelag county, Norway (21). Currently, HUNT Biobank has participated in several large-scale studies that altogether have genotyped ∼34 000 HUNT samples on technologies that include Illumina GWAS, metabo, immuno, exome, and combo chips and exome and whole genome sequencing (22–28). One of the obligations of these studies is to return all the genotype data back to the biobank. We are currently using the EGDMS to organize the data and metadata connected to all these studies. The following illustrates how data and metadata from two HUNT GWAS studies are stored in EGDMS.

**Figure 7.**
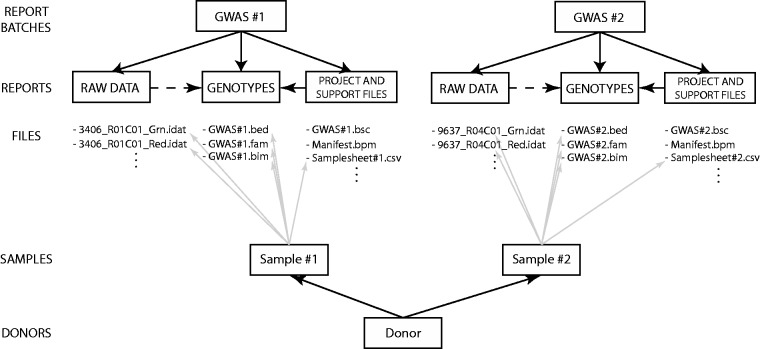
An example of an EGDMS report. Two report batches represent two GWAS experiments. Both report batches use the same report structure, with one report each for raw data, project files and the final product: a report containing genotypes. Each sample is connected to all the files containing any kind of information regarding the sample. Study-specific samples are then connected to donor information.

The structure of EGDMS reports is the same for almost all of the studies, since most of them are GWAS; (Figure 7) illustrates the structure for two GWAS studies. Each report batch, describing a study, contains three reports, corresponding to three logical steps in the process of a study. The first report contains files holding all the raw data; in this case, intensity values originating from Illumina high-density SNP arrays. The second report contains all the support files needed to interpret the raw data, including Genome Studio project files, sample sheets, manifest and cluster files. Last, the third report contains processed genotype data; in these examples, the genotype data are in the PLINK binary PED format (29) (http://pngu.mgh.harvard.edu/∼purcell/plink/binary.shtml). Each study receiving samples from HUNT has study-specific sample identifiers. In the EGDMS report, sample identifiers are connected with any file that contains information about that sample such that sample identifiers are connected with raw data, project files, sample sheets and genotype data (Figure 7). In the end, donor information is connected to the study-specific sample identifiers.

The original data resided on a server with limited access and this data server was different from the EGDMS server. To add the data to the EGDMS server, the egenv command line tool was installed on the data server and run to transfer the metadata to the EGDMS server. A large part of this process was automated by parsing the study sample sheets, which provided information such as sample IDs and unique microarray identifiers. One of the projects added was a study on cardiovascular disease, and that information was added as relevant tags.

**Figure 8.**
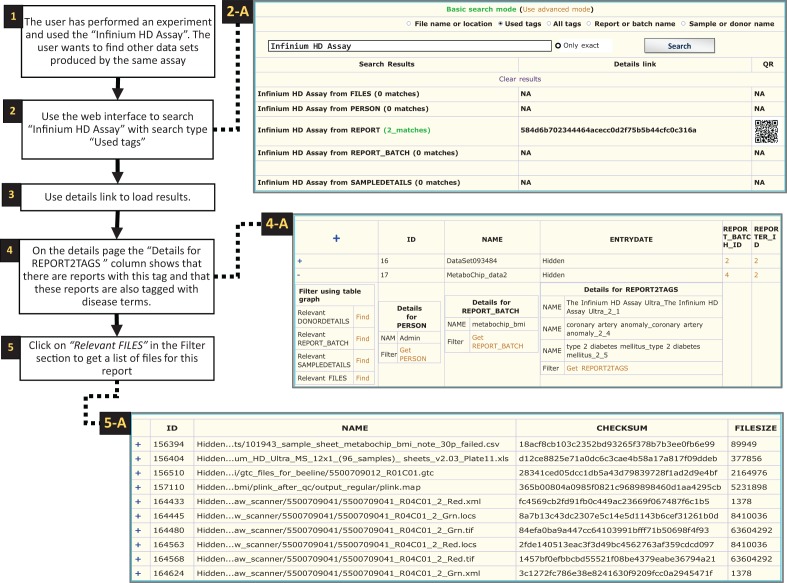
Searching tags. Searching for experiments that have used the ‘Infinium HD Assay’ (1). As the system is not extracting information directly from files, the search results are produced by using the tags. The term ‘Infinium HD Assay’, which is a commercial term used to identify a genotyping assay, was the search query in this case (2). Choosing ‘Used tags’ will search through tags currently used in contrast to all the tags available in the system (as shown in Figure 8). The user opted out the exact match so the search algorithm will look for all tags containing the term ‘Infinium HD Assay’ (e.g. ‘Infinium HD Assay Ultra’). Once the search is finished a results page will be generated as shown in the screen shot (2-A, two reports were found with this tag). Then the ‘Details link’ could be used to display full details of the result (3). On the details page, the reports found are shown along with a link to display a complete list of tags (4). The ‘Find relevant’ links in the ‘Filter using table graph’ column can be used to get lists of files that are related through lineage information (4-A). Files that are related to the specific ‘Infinium HD Assay’ report can thereby be retrieved, even though these file were not directly tagged with the term. In this case, the ‘Relevant FILES’ (5-A) include image files, spreadsheets and assay-related data files.

**Figure 9.**
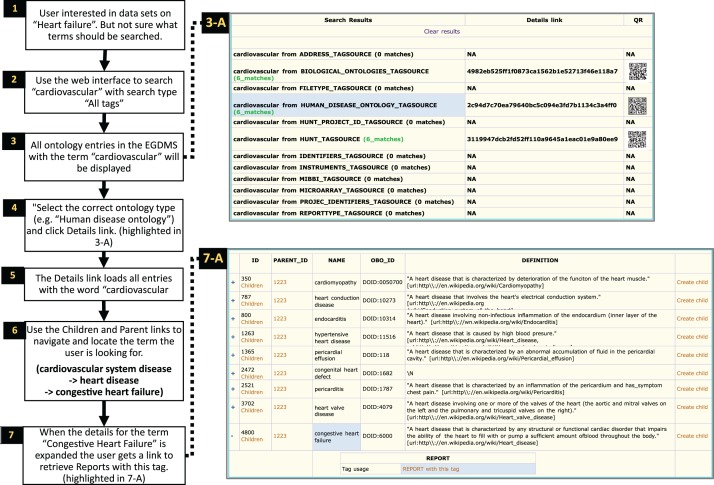
Searching ontology hierarchy. Users unaware of exact controlled vocabulary terms for what they are looking for can search through all tags available in the system. The user in this example is looking for datasets from ‘heart failure’-related studies (1). By providing the term ‘heart failure’ and selecting ‘All tags’ as search criteria (2), the EGDMS finds all occurrences of this term in all tags. The results will contain terms from different tag sources and may also include terms that are currently not used to tag any files (3-A). Browsing through the results, this user selects the tags from the ‘Human disease Ontology tag source’ (highlighted in 3-A) (4). The user can then use ‘Children’ and ‘Parent’ links to explore the tag hierarchy to find if a relevant parent or a child term has been used to tag files (6). In this case, the user navigates to ‘Congestive heart failure’ by selecting child terms of cardiovascular system disease and then heart disease. By expanding (+) to show the details for this term (7, highlighted in 7-A), the user discovers that there is a report that has been tagged with this term. This report can further be explored to locate files and samples grouped under the report (Figure 7).

The web interface provides an overview of the studies, study metadata and access to information on where all the files are physically stored. Figures 8 and 9 illustrate two different ways the web interface can be used to find data. Using the web interface we can, for example, check for the number of participants in each study or find information about the technology used in the studies. The structure described above also makes it easy to do quality controls such as identifying inconsistencies between studies and to export data for specific donors. First, using EGDMS we can identify samples that were used in multiple studies and the files that contain their raw and genotype data. These files can then be analysed to determine to what extent the different studies agree on the genotypes for the same donor. Second, the biobank often get requests for raw data for specified donors. In EGDMS, donor information is connected with the raw data and support files, so we can extract just the raw data for the specific sample and include support files, like SNP array cluster and manifest files. This way, the biobank can easily ship the requested data without including any of the data connected to other samples.

## Discussion

The EGDMS was designed to report the presence of data, including sensitive or private data, and to maintain an extended set of metadata about these data. Importantly, the EGDMS does not store the files themselves, but rather create a catalogue which includes location, provenance, content and lineage information. Therefore, the system can maintain uniform file records transcending file systems, access restrictions, security concerns, geographical locations and formats. These features are well suited for the primary use of this system at the moment, which is to manage data from research conducted on samples from the HUNT Biobank (http://www.ntnu.edu/hunt).

### Comparison with other systems

The main feature of the EGDMS is its ability to locate files by using the extended metadata described in this article. The EGDMS has many advantages over traditional and ad hoc tools.

- EGDMS does not require access to the files when searching.
- The EGDMS can provide a collective view of all related files (grouped using a *report*) from different disconnected locations as a single result.
- The information on *data lineage* and the content descriptions provided by the tagging system is currently only available through the EGDMS.
- Files that are compressed or archived in external storage can be included in searches without having to restore them first.
- The EGDMS caters to the users who do not have the terminal competency to effectively use command line tools such as **find** and **grep**, and do not want to make their own scripts or programs.

There are popular and very good policy-based data management systems like iRODS (https://www.irods.org/) which can provides some of the services of the EGDMS described here. The iRODS system in particular could manage and provide users with a uniform access method for data distributed across heterogeneous systems. The Welcome Trust Sanger institute is using an iRODS solution for managing their high throughput sequencing data and metadata (30). In contrast to iRODS, EGDMS only manages metadata and does not federate access to or host the files themselves. However, compared to iRODS, EGDMS features a richer set of metadata that can capture additional domain-specific metadata and hierarchical and temporal relationships between files and entities. In addition, EGDMS provides a consolidated view of distributed data rather than lists of individual files. Therefore, the EGDMS is a valuable complementing resource for file management systems like iRODS.

Version control systems or source code management systems like CVS (http://ximbiot.com/cvs/), SVN (http://subversion.apache.org/) or git (http://git-scm.com/) are very efficient in managing edits to files. The lineage information of the EGDMS described here should not be confused with such systems. In contrast to recording the different edits to a file, the *data lineage* records what other data are required to generate a certain file and the connections between different transformation states during data processing. For example, lineage captures the connection between raw data and a normalized version of it. Another example is the connection between original experimental data such as sequencing data, additional data such as genomic references or gene annotations used when analysing the data, and results of such analyses such as lists of genetic variants or gene expression levels.

Two other existing systems and strategies to manage genomics metadata are the ISA tools (13) and the eXframe system (31). Moreover, efforts like the Dataverse Network project (http://thedata.org/) and the Dryad Digital Repository (http://datadryad.org/) are successfully hosting and providing methods to share and cite research data. These systems have strengths in data archiving, sharing, integration, standardizsation and visualization, but lack EGDMS’ support for active data acquisition. The EGDMS actively participates in the data collection by collecting file metadata and creating records with default values automatically, by using existing information from the operating system and the file system, and by providing reusable information in the form of tags. More importantly, these systems do not handle the *data lineage* and *provenance *relationships, which are the very root of our EGDMS design. Instead, the ISA tools can complement the EGDMS as they can be used to convert data into standard formats at the end of the collection process. Similarly, the eXframe (31) system can provide a good visualization platform for collected data. Considering all aspects, and especially our requirement for a flexible system for handling provenance and lineage aspects of data, we have developed EGDMS as a new system that complements and can interact with existing systems.

### Installation

Two hurdles when installing and maintaining software systems are dependencies and compatibility issues. The EGDMS addresses these hurdles by minimizing third party dependencies and by using embedded solutions. Specifically, we have selected third party components with a good development base and have some collaboration; for example, the selected application server Glassfish (http://glassfish.java.net/) and the DBMS JavaDB (http://www.oracle.com/technetwork/java/javadb/) are closely linked embedded systems. The advantage of this strategy is that the whole system is packaged as a single unit and ready to run without installing anything else.

### Security

Our main security precaution is the strategy to ‘avoid storing anything worth stealing’. Although access to EGDMS is restricted to registered users, all information gathered by the system could be exposed to the public. Edit privileges, however, require much more control to prevent sabotage and accidental alterations. When activated, two-factor user verification using a mobile phone and email can be used for extra security. The certificate-based authentication used by the egenv tool is suited for terminal operations and avoids the need for the user to type in login credentials all the time. The *diagnose* instruction provided with the egenv tool helps to trouble-shoot and rectify problems if there are legitimate authentication issues.

The system was primarily designed to catalogue research on biobank samples. Such research typically involves several levels of approvals and agreements and the data generated are considered to be sensitive information about the participating individuals. Rather than risk exposing such sensitive data by hosting the data themselves, the EGDMS maintains and exposes the non-sensitive information about the data. This simple solution to a complex issue allows biobanks to advertise and researchers to become aware that the data exists.

### The tagging process

The tagging process described here provides depositors with a method to attach additional information about deposited files and their content in a standardized way and with minimum effort. An alternative to this dynamic process would be for the depositor to fill in a form with all the information upfront. Enforcing depositors to provide standard information in a restricted way will generally result in records having more details, as evident by the record generated by the ISA (13) tool kit, which generates records accordance with the standards such as MIME (15). The downsides with enforcing depositors to provide all information upfront are a higher threshold for depositing files with the system—that is, the more information requested, the less likely users are to routinely deposit their files—and less flexibility in adding to the files new information that were not covered by the forms during the data deposit. The tagging process has relaxed such restrictions to encourage regular use of the system, but this may result in depositors not bothering to provide all the tags necessary to describe the data. Therefore, curators should encourage good practice and make additional edits to complete the records. As users also can create new tags, curators should monitor the new tags and consider structuring these into new or existing ontologies and controlled vocabularies. Importantly, relevant existing data can easily be identified and retagged through the EGDMS system.

When tagging is used effectively, it is easy to integrate the EGDMS with LIMS or sample management systems used in biobanks. The EGDMS can communicate with these systems through static links to the web interface, through the web-services API suing custom build clients, or through wrappers for the egenv tool. Moreover, the QR codes generated for samples and donors can link sample containers directly to the EGDMS and display details about the content by using a scanner.

### Potential uses and future plans

This article’s use-case is the HUNT biobank and the article describes how the EGDMS can be used to catalogue and advertise HUNT’s sensitive data. Specifically, the EGDMS provides a way not only to easily locate data but also maintains the relationships between files and the relationships between the data and the samples used. The current system with open biological ontology and minimum requirements for reporting experiments is tailored to the life science domain. However, the concepts discussed here are valid across research domains and could be used to manage and advertise any type of research data.

With the current setup, the ELIXIR (http://www.elixir-europe.org/) project could benefit from the metadata management concepts discussed here, and the tools are already available to set up a prototype system. The ELIXIR, as a pan-European research infrastructure for biological information, will collect massive amount of research data and a cataloguing system, which can be used for public data and sensitive data alike, is important for easily locating and integrating data. The provenance recording system, which focuses on how the data generated in an experimental context, and the tagging system for data systematic description would provide a standardized interface for the catalogue.

### Known issues with the EGDMS design

As explained above, the relaxed requirements on adding metadata when files are deposited can lead to incomplete entries and thereby reduce the quality of the database content. Another potential problem is missing information about provenance. Provenance can be automatically captured by configuring computational pipelines or workflows to record processing steps and results in EGDMS. However, certain type of provenance, such as connections between data that has been processed by proprietary software or by collaborators off-site, has to be added manually. We do, however, consider the possibility to manually add provenance as one of the key features of EGDMS, as this is essential for our main use-case: the HUNT biobank.

Currently, updates to data already deposited in the system do not happen automatically. A user or a program needs to call the *diff *and *update *operations for each registered location to perform updates. In contrast, configuring a daemon process to monitor files and call update when appropriate would automatically handle updates. The EGDMS, however, was designed to run with minimum privileges without administrator access. In fact, the security module prevents running certain operations as a system administrator. In addition, data kept in a location with no network connectivity with the EGDMS server cannot be automatically checked for changes.

### Technology choices

There could be valid criticisms about the technologies used in the EGDMS system as well. For instance, a hierarchical DBMS rather than the relational one may better handle the *data lineage* information discussed here. Further, much simpler application servers than Glassfish, such as Jetty (http://www.eclipse.org/jetty/), can be used in applications like this. The database manager software was selected considering overall requirements and not just an individual case. Specifically, ability to capture all of the relationships efficiently, documentation, developer community, licence for redistribution, cost, programmatic accessibility, maintainability and interactivity with other third party components were the requirements that influenced decision. The Glassfish server was selected due to its collaboration with JavaDB, its security, its convenient methods for programmatic deployment of web-services, its regular upgrades and its programmatic configurability through available documentation. Having said that, the end-user has the option to replace the DBMS or the application server if desired.

### Ongoing improvements

We have included a tag library with the EGDMS created using publicly available data. This library needs to be expanded by including details from instrument and reagent manufactures, more phenotypic data, disease conditions, file type descriptions and experimental protocols. Special purposes tools need to be included with the system to simplify and to automate the data collection and annotation process. For example, a client to extract tags from sample sheets and from VSF4 files is currently being designed. The current web-service operates on Simple Object Access Protocol (SOAP) and the addition of a Representational State Transfer (REST or RESTful) implementation would introduce more ways to interact with the EGDMS. The only command line client for the web-service is a Java-based tool and more clients and examples for other programming languages will follow. A beta version of the command line client for Microsoft Windows™ is available and will be improved to handle platform-specific issues. The synchronization operation of the system can be improved; for instance, local biobanks can have local servers, which are synchronized with a central server connecting all the biobanks. This way it is possible to have a central information catalogue with less access restrictions containing selected information from the local biobanks. The current system provides support for MySQL as a JavaDB replacement out of the box and more systems including PostgreSQL will be introduced in the future.

## Materials and Methods

The development was carried out using the Netbeans IDE (https://netbeans.org/) on a workstation running Ubuntu 12.04 with Oracle® Java development kit 1.7.0. During development and testing the Glassfish application server (http://glassfish.java.net/) and the MySQL DBMS (http://www.mysql.com/) were used. Java Server Page (JSP) technology, html and Java Scripts (JS) were used to make the web interface. The JDBC API handles the communication with the DBMS and the interfaces. The API for XML Web Services (JAX-WS, http://docs.oracle.com/javase/7/docs/technotes/guides/xml/jax-ws/) with Java Architecture for XML Binding (JAXB) technology (https://jaxb.java.net/) was used to create stub code for the web-service client used with the precompiled version of the egenv tool. The QR code generation was done by using the zxing library (http://code.google.com/p/zxing/). The mail management was implemented using JavaMail™. The user needs to provide a Google mail (Gmail, http://mail.google.com/) account for the mail notifications to work. Bug tracking and developer documentation was maintained using Trac (http://trac.edgewall.org/). The additional data used in the tagging process and shipped with the EGDMS system are listed in Table 1.

**Table 1.** Additional data used in the tagging process and their sources

Type	Source
Minimum Information guidelines from diverse bioscience communities	Downloaded from http://mibbi.sourceforge.net/portal.shtml
Open biological ontologies	Downloaded from http://obofoundry.org/
The Nord-Trøndelag health study (HUNT)^a^	Created using HUNT database export with kind assistance from Jon Heggland.
Sequence Ontology	Downloaded from http://www.sequenceontology.org/

^a^Not available in the public version of EGDMS.

The EGDMS source code is available from https://github.com/Sabryr/EGDMS.

## Supplementary Data

Supplementary data are available at *Database* Online.
